# Asbestos accelerates disease onset in a genetic model of malignant pleural mesothelioma

**DOI:** 10.3389/ftox.2023.1200650

**Published:** 2023-06-27

**Authors:** Pooyeh Farahmand, Katarina Gyuraszova, Claire Rooney, Ximena L. Raffo-Iraolagoitia, Geeshath Jayasekera, Ann Hedley, Emma Johnson, Tatyana Chernova, Gaurav Malviya, Holly Hall, Tiziana Monteverde, Kevin Blyth, Rodger Duffin, Leo M. Carlin, David Lewis, John Le Quesne, Marion MacFarlane, Daniel J. Murphy

**Affiliations:** ^1^ School of Cancer Sciences, University of Glasgow, Glasgow, United Kingdom; ^2^ Department of Respiratory Medicine, Royal Infirmary, Glasgow, United Kingdom; ^3^ CRUK Beatson Institute, Garscube Estate, Glasgow, United Kingdom; ^4^ Glasgow Pleural Disease Unit, Queen Elizabeth University Hospital, Glasgow, United Kingdom; ^5^ MRC Toxicology Unit, University of Cambridge, Cambridge, United Kingdom; ^6^ Centre for Inflammation Research, Edinburgh, United Kingdom; ^7^ Department of Histopathology, Queen Elizabeth University Hospital, Glasgow, United Kingdom

**Keywords:** mesothelioma, asbestos, cancer therapy, GM mouse model, macrophages

## Abstract

**Hypothesis:** Asbestos-driven inflammation contributes to malignant pleural mesothelioma beyond the acquisition of rate-limiting mutations.

**Methods:** Genetically modified conditional allelic mice that were previously shown to develop mesothelioma in the absence of exposure to asbestos were induced with lentiviral vector expressing Cre recombinase with and without intrapleural injection of amosite asbestos and monitored until symptoms required euthanasia. Resulting tumours were examined histologically and by immunohistochemistry for expression of lineage markers and immune cell infiltration.

**Results:** Injection of asbestos dramatically accelerated disease onset and end-stage tumour burden. Tumours developed in the presence of asbestos showed increased macrophage infiltration. Pharmacological suppression of macrophages in mice with established tumours failed to extend survival or to enhance response to chemotherapy.

**Conclusion:** Asbestos-driven inflammation contributes to the severity of mesothelioma beyond the acquisition of rate-limiting mutations, however, targeted suppression of macrophages in established epithelioid mesothelioma showed no therapeutic benefit.

## 1 Introduction

Malignant pleural mesothelioma (MPM) is a devastating neoplasm arising in the lining of the chest cavity that is causally linked to asbestos inhalation (Andujar et al., 2016). This cancer is characterised by a lengthy incubation period between exposure to asbestos and the onset of clinical symptoms, followed by very rapid progression from diagnosis to morbidity (Carbone et al., 2016). Treatment options are severely limited and 5-year survival is <10% (Bibby et al., 2016; Tsao et al., 2018). Although commercial use of asbestos is banned in most western countries, mining and use of asbestos continues unabated in several developing economies. In the UK and elsewhere, a legacy of asbestos-insulated buildings continues to drive new cases of MPM (Blyth and Murphy, 2018).

Direct inoculation of asbestos fibres into rodents has been used to model both early and late stage disease (Bernstein et al., 2010; Chernova et al., 2017; Port and Murphy, 2017). Injection of asbestos into the chest cavity provokes a rapid inflammatory response, comprised initially of neutrophils followed rapidly by macrophage infiltration (Donaldson et al., 1989). Fibre dimensions that thwart engulfment and clearance result in “frustrated phagocytosis” and the establishment of chronic inflammation (Donaldson et al., 2010). The resulting prolonged release of cytokines and reactive oxygen species are thought to create a permissive milieu that drives proliferation and mutation in pleural mesothelial cells (Murphy et al., 2012; Benedetti et al., 2015). Previous longitudinal analysis revealed steadily progressive disease in wild-type mice injected with long-fibre amosite asbestos, with epigenetic silencing of key tumour suppressor loci manifest in pre-neoplastic pleural lesions (Chernova et al., 2017). Progression to mesothelioma however was reported in <10% of such mice after 18 months of incubation and the precise mechanism of progression to malignancy remains poorly understood.

The genetic landscape of end-stage human MPM is now well-defined. Inactivating mutations and deletions of 3 tumour suppressor loci, namely *CDKN2A/B*, *NF2*, and *BAP1* predominate, with functional loss of each tumour suppressor occurring in over 50% of cases and single or combinatorial loss of the 3 loci found in all possible permutations (Bueno et al., 2016; Hmeljak et al., 2018; Nastase et al., 2021; Mangiante et al., 2023). Loss of additional tumour suppressors linked to the Hippo pathway (*LATS1, LATS2, MST1, SAV*), histone methylation (*SETD2, SETDB1*), RNA splicing (*SF3B1, DDX3X*), and mTORC1 regulation (*TSC1, TSC2, ULK2*) occur at lower frequencies. Inactivating mutations of *TP53* are reported to be present in <10% of MPM but are associated with more aggressive disease (Bueno et al., 2016). In contrast, activating mutations and amplification of known driver oncogenes common in other cancer are noticeably absent, although overexpression of *TERT*, linked to mutation of the gene promoter region, has been reported (Tallet et al., 2014).

Genetic deletion of *Trp53* (Marsella et al., 1997), *Nf2* (Fleury-Feith et al., 2003), *Cdkn2a* (Altomare et al., 2011) and *Bap1* (Kadariya et al., 2016) have each been shown to predispose mice to asbestos-induced mesothelioma, albeit with incomplete penetrance and variable latencies. The use of constitutive (i.e., whole organism) and in some instances heterozygous alleles likely masked the true susceptibility of tumour suppressor-deleted pleural mesothelium to carcinogenic transformation. The first purely genetic models of MPM demonstrated that combinatorial loss of multiple tumour suppressors in the appropriate anatomic location sufficed to give rise to MPM in the absence of asbestos inoculation (Jongsma et al., 2008). Intrapleural delivery of Adeno-Cre was used to conditionally delete floxed alleles for *Nf2, Cdkn2a* and *Trp53* in various combinations, and gave rise predominantly to mesothelioma with sarcomatoid or biphasic histology. A somewhat confounding observation was the frequent development of tumours in unintended tissues: these included lymphomas, leiomyomas, tumours of unspecified origin and hepatomegaly. These off-target neoplasms suggest escape of the viral vector from the chest cavity with consequent Cre-mediated allele inactivation in the affected tissues. More recently, the same strategy was applied to mice bearing combinations of floxed alleles for *Nf2*, *Cdkn2a* and *Bap1* (Kukuyan et al., 2019; Badhai et al., 2020). Although Cre-mediated deletion of any pair of homozygous alleles sufficed to give rise to pleural mesothelioma, acquired loss of BAP1 protein expression was found in the majority of *Nf2* ^*fl/fl*^ *;Cdkn2a* ^*fl/fl*^ tumours *in vivo*, and homozygous deletion of all three genes was required for spheroid formation *in vitro* (Kukuyan et al., 2019). As reported for the *Trp53* study (Jongsma et al., 2008), tumour histology was predominantly sarcomatoid (Kukuyan et al., 2019) or biphasic (Badhai et al., 2020) and off-target tumours were again frequently detected in both studies.

Such *in vivo* studies unequivocally demonstrate a functional requirement for these tumour suppressors to prevent mesothelioma development. One question they fail to address however is whether asbestos-driven inflammation plays any role in MPM progression beyond its contribution to the acquisition of rate-limiting mutations. This question has immediate clinical relevance, as the persistence of asbestos fibres sustains an inflammatory microenvironment, even after the acquisition of disease-limiting mutations and throughout therapeutic intervention. We therefore asked if intrapleural injection of disease-relevant doses of asbestos (Bernstein et al., 2010) has any impact on disease progression in mice that are genetically destined to develop MPM. Our results show a dramatic acceleration of disease onset in such mice upon inclusion of asbestos exposure, with significant implications for pre-clinical modelling of this disease *in vivo.*

## 2 Materials and methods

### 2.1 Mice and procedures

The *Nf2* floxed (Giovannini et al., 2000), *Tp53* floxed (Marino et al., 2000) and *Cdkn2a* knockout (Krimpenfort et al., 2001) mice were described previously. All experiments involving mice were approved by the local ethics committee and conducted in accordance with UK Home Office licence numbers PE47BC0BF, 70/7950 & 70/8646. Experiments were performed and reported in accordance with the ARRIVE guidelines. Mice were housed in a constant 12h light/dark cycle and fed/watered *ad libitum*. Both males and females were included in approximately equal numbers and mice were randomly assigned to induction or treatment cohorts, balanced only for sex. Recombinant lentivirus expressing CRE recombinase was purchased from the University of Iowa vector core facility. Allelic recombination was induced as previously described (Grosso et al., 2021): administration of lentiviral vector carrying CRE recombinase was performed on mice aged 8–10 weeks via single intrapleural injection of 10^7^ viral particles per mouse. Where indicated, asbestos (long fibre amosite; Poland et al., 2008)) was administered via single intrapleural injection of 25 μg fibres per mouse 10 days post CRE administration. CSF1R inhibitor (AZD7507) was provided by Astra Zeneca under a research collaboration agreement with CRUK Beatson Institute. AZD7507 was dissolved in 0.5% HPMC + 0.1% Tween80 in dH2O and administered at 100 mg/kg twice daily by oral gavage. Cisplatin (MedChemExpress #HY-17394) at 2 mg/kg and Pemetrexed disodium heptahydrate (Sigma-Aldrich #SML1490) at 50 mg/kg were administered in combination (IP injection) in two rounds, separated by 15 days interval, with or without 5 days AZD7507 (100 mg/kg twice daily) treatment immediately preceding each round of chemotherapy. Mice were given Dulbecco’s Phosphate Buffered Saline (DPBS) from Gibco (#D8537) supplemented with D-Glucose (Sigma-Aldrich #G7021) at 3 g/L for 2–3 days post chemotherapy to alleviate adverse reaction. Routine health monitoring was performed by facility personnel without knowledge of experimental details. Humane end points were defined as exhibition of 2 or more symptoms: weight loss, elevated breathing, hunching, untidy coat. All mice were sacrificed using a schedule 1 procedure.

### 2.2 Histology and immunohistochemistry

For histological examination, tissue was harvested, fixed overnight in formalin and embedded in paraffin for sectioning, followed by standard staining in hematoxylin and eosin (H&E). Tumour pathology was determined by an experienced translational thoracic pathologist (JLQ). For manual IHC staining for Ki67 (Fisher Scientific, RM-9106-S0, 1:1000), FFPE tissue sections were deparaffinised in 3 changes of xylene and rehydrated in graded ethanol solutions. Antigen retrieval was performed by microwaving in 10 mM Sodium Citrate, pH6.0. Endogenous peroxidases were quenched in 3% H2O2 and non-specific binding was blocked with 1 or 3% BSA solution. Staining for all other antibodies (see table below) was performed by Leica Bond Rx or Dako autostainers: Sections were dewaxed and antigen retrieval was performed using solution ER2 (Leica) for 20 min at 95°C or Enz1 (Leica) for 10 min at 37°C. For Dako autostaining, pH6 antigen retrieval was performed off-board in a Dako PT module using the pH6 retrieval buffer for 20 min at 97°C. High pH TRS (pH9) was done off-board in a Dako PT module using the high pH retrieval buffer for 20 min at 97°C. Liquid DAB was used as chromogen in all cases. Detailed antibody information and staining conditions are as follows:

**Table:** 

Ab	Clone	Company	Code	Autostainer	Retreival	Dilution
B220/CD45R	RA3-6B2	Abcam	Ab64100	Leica Bond Rx	ER2 20 min	1:200
CD4	4SM 95	eBioscience	14-9766-82	Leica Bond Rx	ER2 20 min	1:500
CD8	4SM15	eBioscience	14-0808-82	Leica Bond Rx	ER2 20 min	1:500
Cytokeratin (Pan)	AE1/AE3	Thermo	MS-343	Dako Autostainer Link48	pH6 20 min	1:100
F4/80	CI:43-1	Abcam	Ab6640	Leica Bond RX	Enz1 10 min	1:100
LY6G	IA8	BioXcell	BE0075-1	Leica Bond RX	ER2 20 min	1:60,000
NKP46/NCR1		R &D systems	af2225	Dako Autostainer Link48	High pH TRS	1:200
ciSMA	144	Sigma-Aldrich	A2547	Dako Autostainer Link48	pH6 20 min	1:25000
Vimentin	D21H3	Cell Signalling	5741	Dako Autostainer Link48	High pH TRS	1:100
WT1	[CAN- R9(IHC)-56- 21	Abcam	Ab89901	Dako Autostainer Link48	High pH TRS	1:250

### 2.3 IHC quantification

Quantification was performed on IHC stained slides using QuPath or Halo open source digital pathology software (Bankhead et al., 2017). Tumour regions were annotated manually followed by automated cell detection analysis. For F4/80 staining, accurate cell counts could not be obtained using QuPath and data are presented as area of tumour stained positively. Graphical representation of data was performed using GraphPad-Prism.

### 2.4 Flow cytometry

Cells from pleural lavages were collected and split into two wells to be stained for flow cytometry as follows. After red blood cell lysis, cells were stained with ZombieNIR (Biolegend, 423106) and then blocked with TruStain FcX (Biolegend, 101320). Next, cells were incubated with fluorescently conjugated Abs diluted in Brilliant Stain Buffer (BD, 566349) and fixed in 2% formaldehyde in PBS (Thermo, 28908). Before analysis using a BD Fortessa flow cytometer, counting beads (Spherotech, Catalog # ACFP-70-10) were added for quantification. Data were analysed with FlowJo software V.10. (BD). Leukocytes (Single cells ZombieNIR^−^CD45^+^) were further gated using panel 1 as eosinophils (CD11b^+^SiglecF^+^), Ly6C^hi^ monocytes (SiglecF^−^CD11b^+^CD115^+^F4/80^-^Ly6C^hi^), macrophages (SiglecF^−^CD11b^+^CD115^+^F4/80^+^) and neutrophils (SiglecF^−^CD11b^+^CD115^-^Ly6G^+^); and panel 2 as B cells (CD19^+^CD3^-^), γδ T cells (CD19^−^CD3^+^TCRD^−^), CD8 T cells (CD19^−^CD3^+^TCRD^−^CD8^+^), CD4 T cells (CD19^−^CD3^+^TCRD^−^CD8^−^CD4^+^) and NK cells (CD19^−^CD3^−^TCRD^-^CD8^−^CD4^−^NKp46^+^). Antibodies used for FACS analysis, Panel 1: CD115-BV421 AFS98 Biolegend 135513; CD11b-BV650 M1/70 Biolegend 101259; CD45-BV711 30-F11 Biolegend 103147; F4/80-FITC Cl:A3-1 BIO-RAD MCA497F; Ly6C-Percp/Cy5.5 HK1.4 Biolegend 128012; Ly6G-BUV395 1A8 BD Bioscience 563978; SiglecF-AF647 E50-2440 BD Bioscience 562680. Panel 2: TCRD-FITC GL3 Biolegend 118105; CD19-BV605 6D5 Biolegend 115540; CD3-Percp/Cy5.5 145-2C11 Biolegend 100328; CD4-PE/CY7 RM4-4 Biolegend 116016; CD8-BUV395 53-6.7 BD Bioscience 563786; NKp46-BV421 29A1.4 Biolegend 137612; CD45-BV711 30-F11 Biolegend 103147.

### 2.5 *In Vivo* imaging

Human magnetic resonance imaging (MRI) protocols were adapted to mouse models to support development of non-invasive imaging endpoints. In the initial pilot phase, to determine whether pleural effusion could be detected by MRI, wildtype (WT) mice were injected with a known intra-pleural volume of minimal essential media (MEM). In the subsequent expansion phase, further MRI imaging features of pre-terminal CNP mice were characterised. MRI was performed on nanoScan^®^ (1T) scanner (Mediso Ltd, Budapest, Hungary). Prior to imaging, mice were anaesthetised with isoflurane for induction at a rate of 5% (v/v) and were maintained under inhaled isoflurane anaesthesia via nose cone 2%–2.5% (v/v) in 95% oxygen during the entire imaging procedure duration. During the procedure, body temperature was maintained by an external heat source and respiratory rate was monitored by a pneumatic pad via the inbuilt “Mediso mouse monitoring system”. All animals were scanned by using described settings and parameters. After preliminary scout images, whole body T1 and T2-weighted coronal/sagittal MRI sequences as well as axial MRI sequences were acquired. The MRI parameters were as follows: for T1 GRE 3D coronal/sagittal imaging, number of slices 60, slice thickness 0.5 mm, field of view 100.0 mm × 35.0 mm, Echo time (TE) 3.8 m s, repetition time (TR) 20 m s, Flip Angle 30°, number of excitations 10 and for T2 FSE 3D imaging, number of slices 50, slice thickness 0.5 mm, field of view 42.0 mm × 42.0 mm, Echo time (TE) 63.7 m s, repetition time (TR) 2000 m s. During the pilot, wild type mice (n = 1 each) received an intra-pleural injection of 500 μl or 1000 μl normal saline. In the expansion phase, 8–10-week-old mice received an intra-pleural injection of 10^7^pfu of lentiviral vector expressing Cre-recombinase, followed 10-days later by 25 μg of long-fibre amosite asbestos. Timing of pre-terminal imaging was dictated by symptoms. The images were analysed using Vivoquant 4.0 software (Invicro, MA, United States). Pleural effusion and visible tumour were subjectively scored, compared by genotype and quantified by volumetric segmentation. PET/MR imaging was based on a published protocol (Witney and Lewis, 2019). In brief, we anaesthetised and maintained mice using 1%–3% isoflurane in 2 L/min oxygen before cannulating the lateral tail vein. Mouse body temperature was maintained at 37°C using an external heat source prior to and throughout anaesthesia and imaging. A bolus intravenous injection of 200 µl [18F]-FDG (41.71 ± 9.9 MBq), diluted in 0.9% saline solution, was administered via the tail vein cannula. We acquired proton T2-weighted MR images (T2 FSE 3D axial MR, resolution 0.13 × 0.13 × 0.5, matrix size 168 × 160 × 150, repetition time (TR) 2000 m s, Echo time (TE) 54.6 m s, Flip Angle 90°, number of excitations 2), in the prone position during an anaesthetised uptake phase of 50 min, followed by a PET scan at 50–60 min using a NanoScan PET/MRI (1T) scanner (Mediso Ltd, Budapest, Hungary). PET images were corrected for decay, attenuation, scatter, deadtime and random coincidences and reconstructed (3D iterative static reconstruction, matrix size 105 × 105 × 237, with isotropic 0.4 mm voxels) using 3D Tera-Tomo (Mediso Ltd, Budapest, Hungary). Images were visualised using Vivoquant version 3.0 (InviCRO, MA, United States). Immediately following PET, mice were euthanized by CO2 inhalation. A small incision was made in the trachea and a blunt 19G cannula (Western laboratory service Ltd, Horndean, UK) was inserted. A 90% OCT/10% PBS mix was drawn into a 5 ml syringe which was then slowly dispensed into the lungs. Once the lungs were fully inflated, the cannula was removed and the jugular vein was cut to allow for blood expansion during freezing. The carcass was then placed in 2-methybutane (Sigma- Aldrich, MO, United States) which had been cooled for 5 min on dry ice. Once frozen, the fur was removed and the torso was isolated and mounted on a specimen disc, using Optimal Cutting Temperature (OCT) solution (Tissue-Tek, CA, United States) for cutting on a Leica CM3050 S cryostat (Leica Biosystems, Wetzlar, Germany). Sections were cut at 10 μm and mounted on PolyFrost Poly Lysine Coated Adhesive Frosted slides (Solmedia Ltd, Shrewsbury, UK) which were then placed face down on a phosphor screen BAS-IP SR 2 (GE Healthcare Lifescience, MA, United States), and left overnight in an exposure cassette (GE Healthcare Lifescience, MA, United States). The following day, phosphor screens were scanned using a Typhoon FLA 7000 IP imager (GE Healthcare Lifesciences, MA, United States) using a 635 nm excitation laser and an IP (390 BP) filter. ImageJ (National Institutes of Health, MD, United States) was used to visualize the autoradiography sections. We stained the sections with H&E and scanned them using a Leica SCN400F slide scanner (Leica Biosystems, Wetzlar, Germany) and aligned these images to the autoradiograms acquired from the Typhoon.

### 2.6 RNA sequencing

Pleural tumours were harvested without fixation and flash frozen for RNA-Sequencing. Total RNA isolation was performed using Trizol according to a modified manufacturer’s protocol: after the isopropanol precipitation step, RNA pellets were resuspended in 100 µl H_2_O. 100 μl acid phenol: chloroform (Ambion) was added, samples were vortexed for 20 s and spun at 13,000 rpm for 10 min. The aqueous phase was extracted with 100 µl of chloroform and spun at 13,000 rpm for 3 min. The aqueous phase was precipitated with 2 µl glycogen, 10 µl NaAc and 300 µl EtOH and pellets were then stored at −20°C for 24–48 h. Pellets were washed with 500 µl 75% EtOH, spun again at 13,000 rpm for 5 min. Pellets were allowed to air dry, then resuspended in 20 µl H_2_O (milliQ). RNA concentrations were obtained using a NanoDrop One (Thermo Fisher Scientific). RNA integrity was assessed using RNA ScreenTape (Agilent Technologies) according to the manufacturer’s protocol using a 2200 TapeStation System (Agilent Technologies). A RIN value > 7 was deemed acceptable. cDNA was generated using TruSeq stranded mRNA library prep kit (Illumina) followed by NextSeq500 High output 75 cycle sequencing. FASTQ files were aligned to the mouse genome (GRCm38.93) using HiSat2 and TopHat2 (Kim et al., 2015). Expression levels were determined and statistically analysed by a workflow combining HTSeq (Anders et al., 2015), the R environment (R Core Team, 2021), utilising packages from the Bioconductor data analysis suite (Huber et al., 2015) and differential gene expression analysis based on the negative binomial distribution using the DESeq2 package (Love et al., 2014). Pathway analysis was performed using MetaCore GeneGO (Clarivate).

### 2.7 Statistical analysis

Quantitative data were uploaded into Prism spreadsheets for analysis and graphic production. Statistical significance was determined by unpaired T-test or ANOVA with *post hoc* Tukey test (ns = not significant; *p* > 0.05, *; *p* < 0.05, **; *p* < 0.01, ***, *p* < 0.001; ****, *p* < 0.0001). For Kaplan-Meier plots, the Mantel Cox logrank test was performed.

## 3 Results

### 3.1 Asbestos exacerbates genetically driven mesothelioma

**FIGURE 1:**
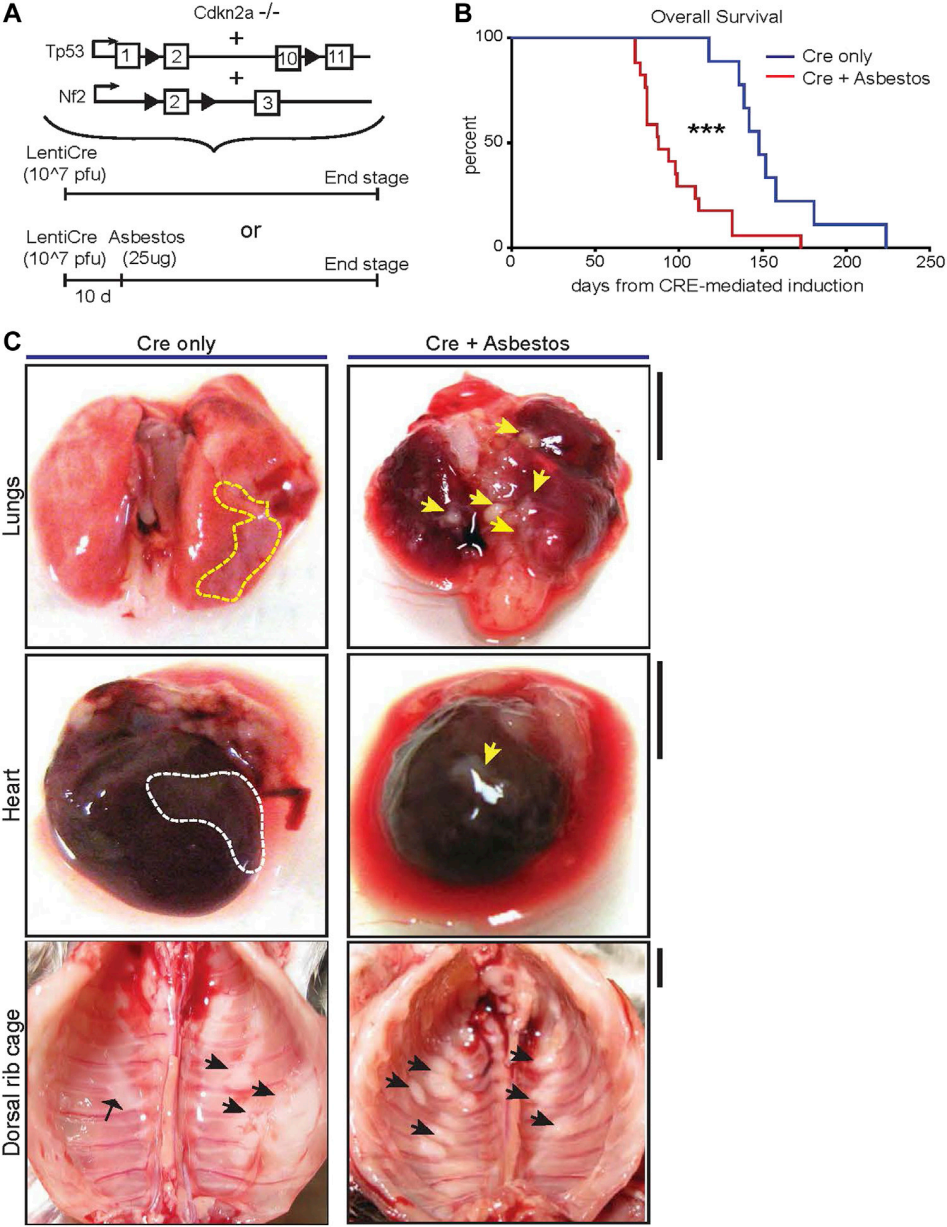
Asbestos injection reduces survival of CNP mice **(A)** Schematic of the alleles and induction strategies employed in this study. Black arrows deonte LoxP sites. **(B)** Kaplan Meier plot of overall survival of lenti-Cre induced CNP mice with (N = 17) or without (N = 9) asbestos injection. *** denotes *p* < 0.001 (Mantel Cox logrank test). **(C)** Photographs showing representative examples anatomic distribution of visible MPM lesions (arrowheads) in mice from **(B)**. Dashed lines circumscribe areas of lateral mesothelioma growth; arrowheads indicate individual mesothelioma tumours. Scale bars = 4 mm.

To investigate the impact of asbestos on mesothelioma progression in mice that are genetically destined to develop mesothelioma, we used triple allelic mice previously described to be sufficient for mesothelioma development in the absence of fibre exposure: *Cdkn2a* nullizygous mice bearing homozygous floxed alleles of *Nf2* and *Trp53* (*Cdkn2a* ^*−/−*^ *;Nf2* ^*fl/fl*^ *;Trp53* ^*fl/fl*^–abbreviated as CNP) (Jongsma et al., 2008). To initiate tumours in the appropriate anatomic location (i.e., the pleural cavity), adult mice triple homozygous for all 3 alleles were injected intrapleurally with 10^7^ pfu of lentiviral vector expressing Cre recombinase. After a 10-day interval to minimise the potential for viral infection of infiltrating leukocytes, mice were similarly injected once with low dose (25 μg) long fibre amosite asbestos or left untreated (Figure 1A). Mice were monitored for development of clinical symptoms and euthanised for tissue collection at pre-defined humane end-points. CNP mice induced with lenti-Cre all developed clinical symptoms, notably including hiccups and laboured breathing. Strikingly, mice that were also administered asbestos exhibited dramatically accelerated disease, requiring euthanasia at a median of 88 days post Cre-mediated induction, as compared with those that did not receive asbestos, which had a median survival of 148 days post induction (Figure 1B). Asbestos administration to uninduced CNP mice did not result in any mice developing symptoms of disease within the same timeframe (data not shown), in agreement with previous reports of similarly low dose asbestos treatment of wild-type mice (Chernova et al., 2017). Upon dissection, frank tumours were clearly visible on the mesothelial surfaces of the lungs, heart, diaphragm and chest walls of all lenti-Cre induced mice, consistent with presentation in human mesothelioma patients. Tumour burden was visibly more pronounced in mice that also received asbestos compared with those that did not (Figure 1C). Notably, off-target tumours in other tissues, such as those reported in similar allelic mice induced using high doses of adenoviral-Cre vectors (Jongsma et al., 2008; Kukuyan et al., 2019; Badhai et al., 2020), were not detected, either in the presence or absence of asbestos exposure.

### 3.2 Histological characterization of lenti-Cre and/or asbestos induced lesions

**FIGURE 2:**
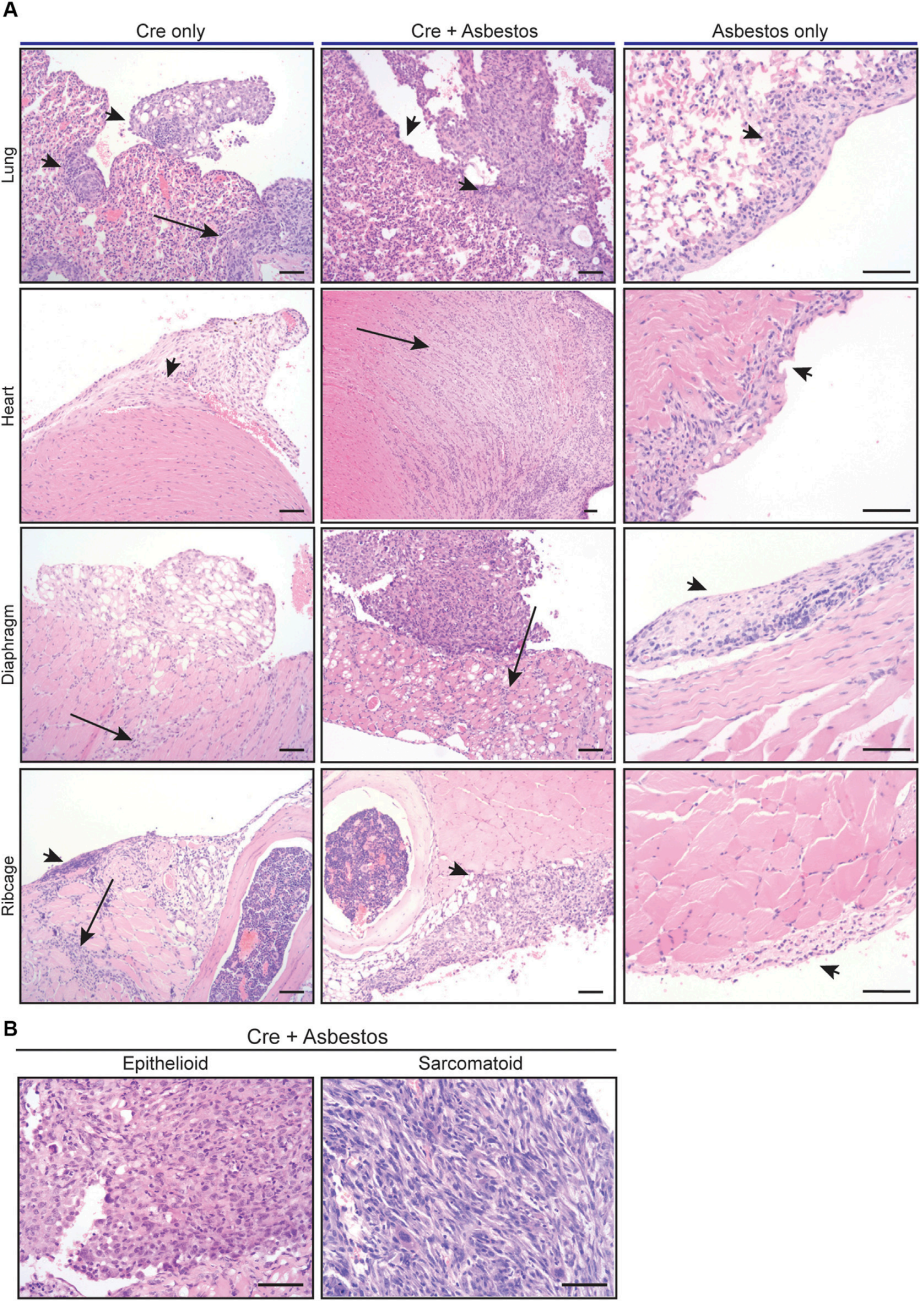
Histology of mesothelial lesions in CNP mice induced with lenti-Cre and/or asbestos **(A)** Representative images of indicated H&E stained tissues from mice induced with lenti-Cre (N = 9), Cre + asbestos (N = 17), both at end-stage, or asbestos without Cre-mediated allele inactivation, harvested 10 months after asbestos injection (N = 3). Arrows indicate neoplastic disease; arrowheads indicate reactive mesothelium. Scale bars = 100 μm. **(B)** H&E-stained examples of epithelioid and sarcomatoid histologies of tumours from CNP mice induced with lenti-Cre + asbestos. Scale bars = 100 μm.

Harvested organs presenting with visible disease were sectioned and stained with H&E for histological examination. The visceral and parietal pleura of uninduced CNP mice injected with asbestos alone and harvested 10 months later all showed discrete regions of reactive thickening, reflective of chronic inflammatory disease, but no tumour formation. In contrast, histological examination confirmed the presence of malignancy in all CNP mice induced with lenti-Cre. Tumours arising in mice induced with Cre alone tended to grow laterally and often contained cystic or luminal structures, whereas those induced by the combination of Cre + asbestos typically formed densely packed spheroids, often with a necrotic core. Marked invasion into cardiac muscle and diaphragm was evident in both tumour-prone cohorts but was more commonly observed in mice that received the combination of Cre + asbestos (Figure 2A). Human MPM is a heterogenous disease with three main histological subtypes, depending on the predominant cellular component and biological behaviour. The epithelioid subtype accounts for 50%–70% of cases, 10%–20% of cases are sarcomatoid, and biphasic accounts for approximately 30% of reported cases (Rossini et al., 2018; Nicholson et al., 2020). Tumours arising in CNP mice were predominantly (>90%) epithelioid with a small subset of lesions showing discrete regions of sarcomatoid morphology (Figure 2B).

### 3.3 Asbestos does not overtly alter the tumour-cell intrinsic mesothelioma phenotype

**FIGURE 3:**
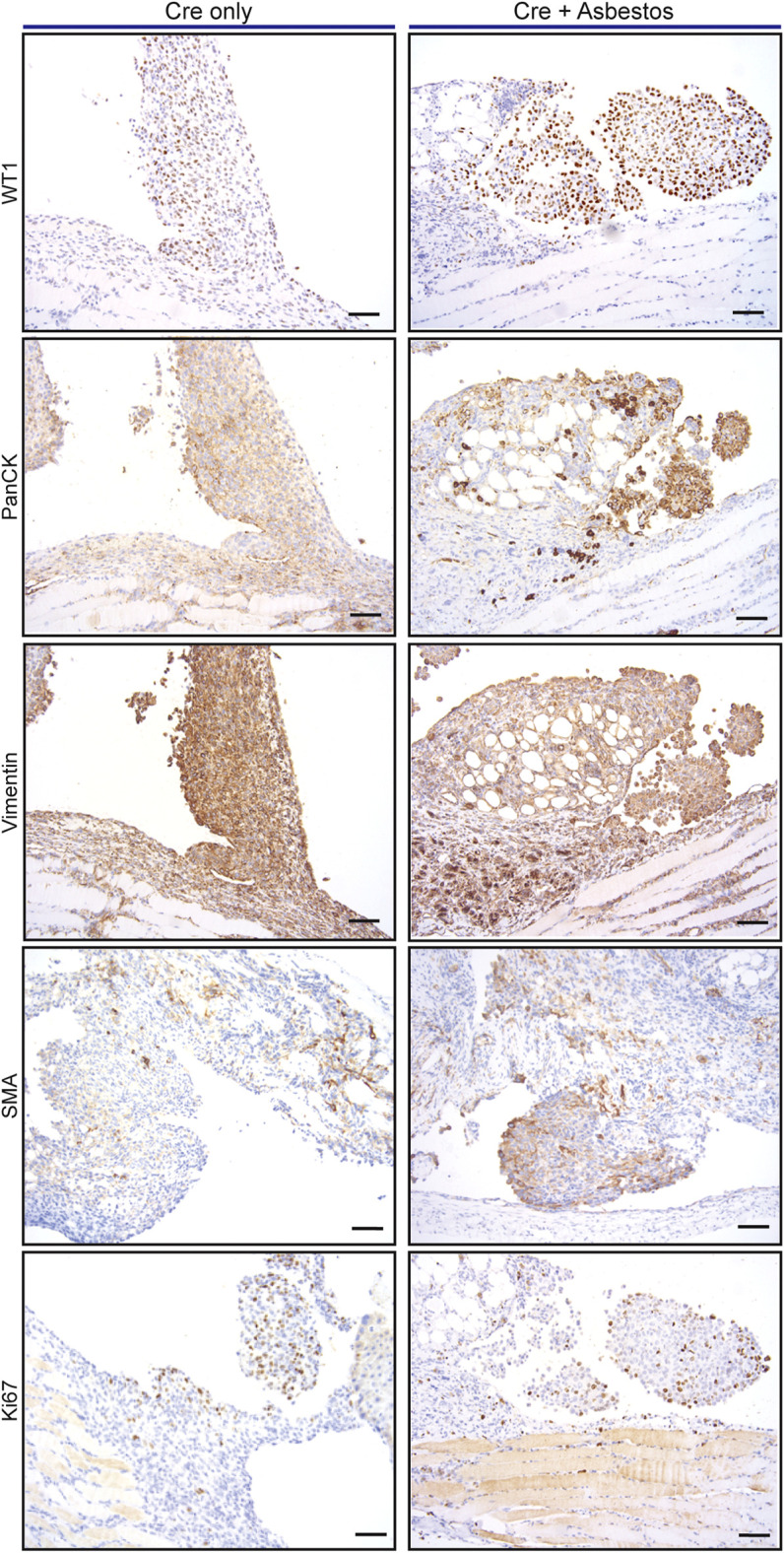
Asbestos does not alter the tumour phenotype of induced CNP mice A) Representative IHC staining of tumours arising from diaphragm mesothelium in CNP mice induced by lenti-Cre, with and without asbestos. Scale bars = 100 μm.

We asked if the inclusion of asbestos manifestly altered the tumour cell-autonomous characteristics of mesothelioma arising in the CNP mouse model. For this analysis we focused on tumours arising from the diaphragm mesothelium, given that decalcification protocols required for analysis of the chest wall can adversely impact immunohistochemical staining. It should be noted that tumours arising on the diaphragm were histologically indistinguishable from those arising on the lung or mediastinum. Tumours of approximately equal size arising in lenti-Cre induced mice with and without asbestos were compared by IHC for expression of cell lineage markers (pan-cytokeratin, vimentin and smooth muscle actin) along with Ki67 and the commonly used mesothelioma markers WT1 and Mesothelin. Individual tumours stained positively for all such markers to varying degrees, however averaging IHC marker expression across tumours in individual mice showed no consistent difference in expression of WT1 or Ki67, suggesting that the inclusion of asbestos does not overtly influence the tumour phenotype (Figure 3; Supplementary Figure S1).

### 3.4 Asbestos dramatically increases macrophage infiltration of tumours

**FIGURE 4:**
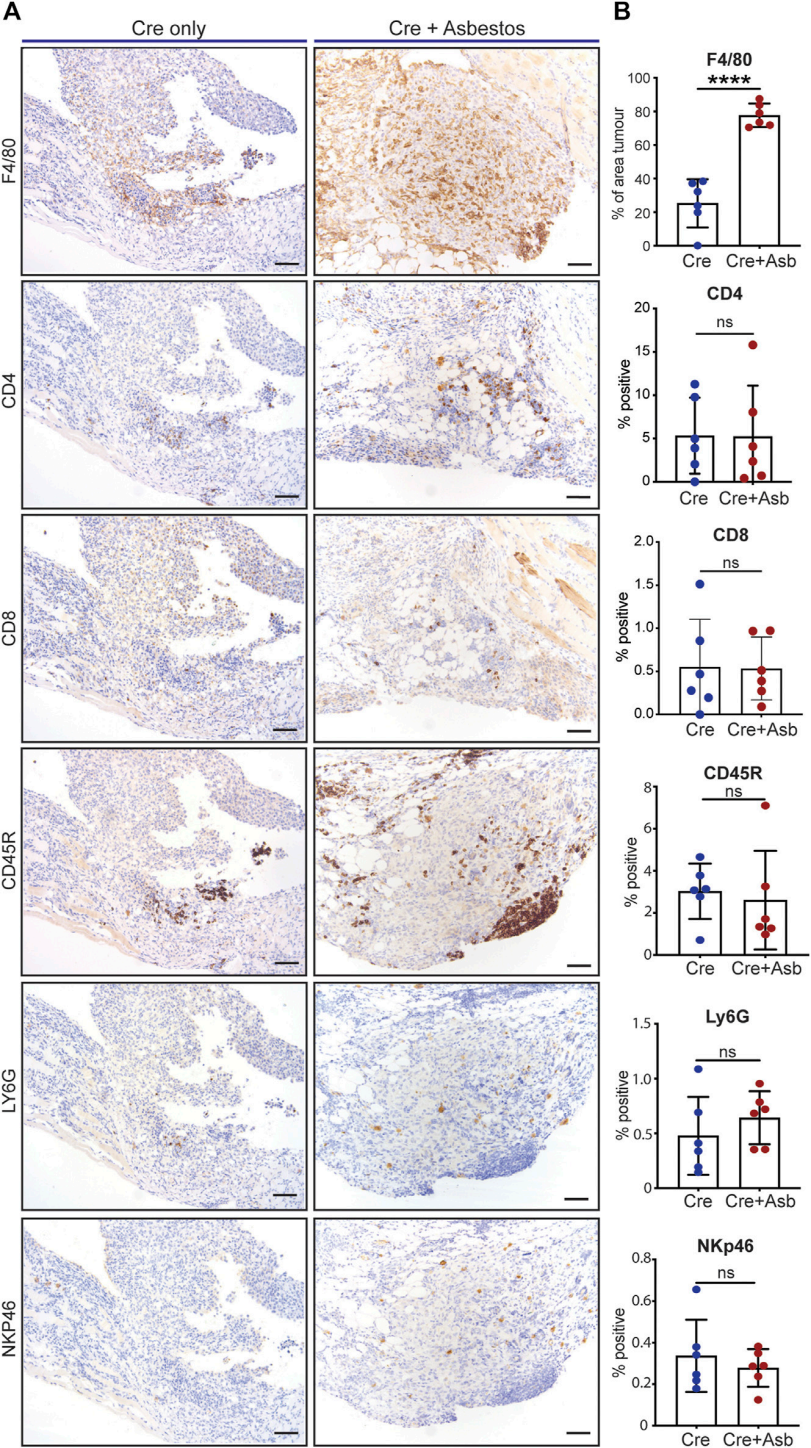
Asbestos drives increased macrophage infiltration in CNP tumours **(A)** Representative IHC images show detection of tumour infiltration by major leukocyte cell types in CNP mice induced with lenti-Cre with and without asbestos. Scale bars = 100 μm. **(B)** Quantification of tumour-infiltrating leukocytes as per **(A)** in 6 mice from each cohort. **** denotes *p* < 0.0001 (T-test); ns = not significant.

Human pleural mesothelioma is characterised by a pronounced immunosuppressive inflammatory microenvironment (Lee et al., 2018; Patil et al., 2018; Blum et al., 2019; Klampatsa et al., 2019; Creaney et al., 2022; Kosari et al., 2022). Recent work has demonstrated the presence of all major immune populations in both pre-malignant lesions of asbestos-exposed wild-type mice (Chernova et al., 2017) and in end-stage tumours of GE mice without asbestos exposure (Badhai et al., 2020). We therefore investigated the impact of asbestos on major tumour-infiltrating immune populations in the CNP mouse model. End-stage tumours on the diaphragms of CNP mice induced with Cre +/- asbestos were stained for expression of CD4 (helper T cells), CD8 (effector T cells), CD45R (aka B220, expressed on B lymphocytes), NKp46 (NK cells), Ly6G (granulocytes, primarily neutrophils) and F4/80 (macrophages) to identify tumour-infiltrating leukocytes. Note that tertiary lymphoid structures, readily discernible adjacent to multiple lesions from both tumour-prone cohorts, were omitted from scoring to avoid skewing quantification. Whereas most tumour-infiltrating immune populations showed no quantitative difference between cohorts treated with or without asbestos, F4/80-positive macrophages were consistently and dramatically increased in tumours from asbestos-treated mice (Figures 4A, B).

### 3.5 Detection and analysis of progressive pleural effusion in CNP mice

Suspected cases of human MPM commonly first present with pleural effusion but a relatively low percentage of these progress to MPM within 1–2 years (Blyth and Murphy, 2018; Asciak et al., 2021). Pleural effusions are a rich source of inflammatory and effector immune populations and can also harbour large numbers of viable tumour cells (Chernova et al., 2016). Similar to human MPM patients, pleural effusion was present in end-stage Cre-induced CNP mice and was more frequent in mice that received asbestos than those that did not. Pleural effusion is readily detectable by magnetic resonance imaging (MRI) and MRI has moreover proven superior to CT for volumetric analysis of pleural disease (Tsim et al., 2020; Katz et al., 2023). To test if MRI could be used to reliably detect pleural effusion in our mouse model, we injected wild-type (tumour-free) mice with defined volumes of minimal essential media (MEM). 500 μl and 1 ml of intrapleurally injected fluid was readily visible by MRI, as indeed was spontaneously arising pleural effusion in end-stage CNP mice (Supplementary Figure S2A). We next used [^18^F]-Fluorodeoxyglucose positron emission tomography-coupled MRI (FDG-PET-MRI) and found CNP tumours to be variably glucose avid with modest uptake of labelled probe by a subset of histologically visible tumour lesions (Supplementary Figures S2B, C).

Genetically engineered mouse models offer tremendous potential for investigating early disease progression, yielding unparalleled insight into the possible course of pre-symptomatic human disease. With this in mind, we investigated the immune microenvironment of the pleural cavity in CNP mice induced to develop mesothelioma, well in advance of symptom onset. To normalize for fluid recovery between mice, we used intrapleural injection of 1 ml MEM to perform a pleural lavage, and labelled cells in the recovered volumes with 2 separate antibody panels to identify specific myeloid or lymphoid cell lineages, respectively. Pleural lavage samples were collected from mice induced with lenti-Cre + asbestos at 30, 60, and 75 days post induction and from mice induced in the absence of asbestos at day 60 post induction. Flow cytometry was used to measure each major leukocyte cell population present in the pleural cavity. The results showed a progressive increase in leukocyte infiltration over time, with a significant rise in B, CD8^+^, CD4^+^ and γδ T cells, along with small cavity macrophages, between 60 and 75 days post induction, and a similar trend evident in other lineages (Supplementary Figure S3). These data suggest a dynamic relationship of nascent mesothelioma tumours with the immune cells of the pleural cavity.

### 3.6 Single-agent suppression of macrophages fails to impact survival

**FIGURE 5:**
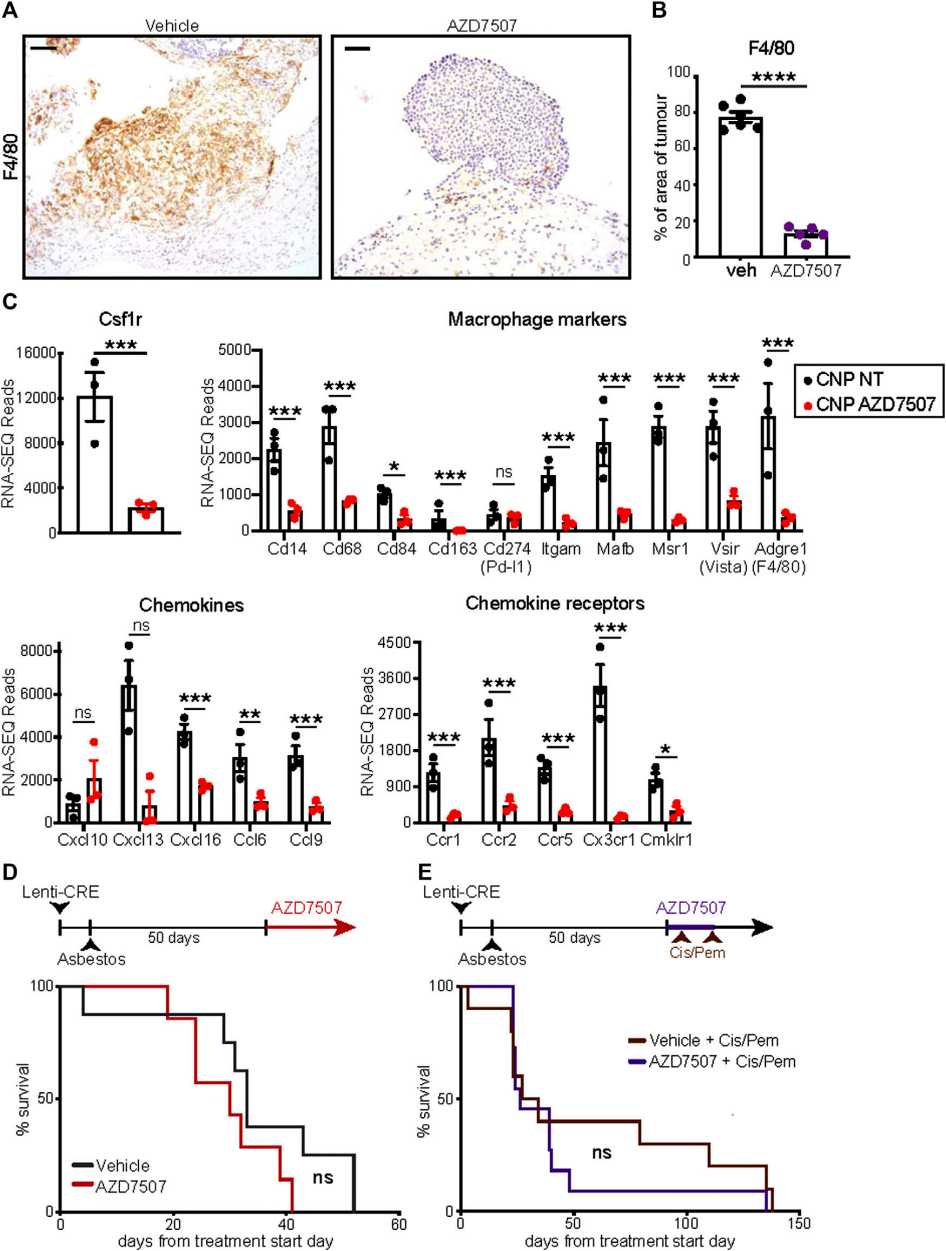
Suppression of macrophages fails to impact survival of CNP mice **(A)** IHC validation of macrophage suppression *in situ* by AZD7507. Left panels show representative images of F4/80-stained diaphragm tumours from end-stage CNP mice induced with lenti-Cre + asbestos and treated with AZD7507 (right) or vehicle control (left). Scale bar = 100 μm. **(B)** Quantification of diaphragm tumour F4/80 staining in mice treated with AZD7507 (N = 5) or vehicle (N = 6). Mean ± SEM shown. **(C)** Normalised RNA-SEQ read counts for selected genes, grouped by function, from end-stage tumours of CNP mice treated with or without AZD7507 from day 60 post-induction (N = 3 per cohort). Individual values, Mean and SEM shown. Adjusted *p* values (T-test) were calculated in R: *** denotes *p* < 0.001; ** denotes *p* < 0.01; * denotes *p* < 0.05; ns = not significant. **(D)** Kaplan Meier plot of overall survival of mice treated with AZD7507 (N = 7) or vehicle control (N = 8) from 60 days post tumour initiation. Ns = Not significant (Mantel Cox logrank test). **(E)** Kaplan Meier plot of overall survival of mice treated with 2 rounds (brown arrows) of Cisplatin + Pemetrexed (Cis/Pem) with (N = 12) or without (N = 10) AZD7507. Ns = Not significant (Mantel Cox logrank test).

Given the pronounced increase in tumour infiltrating F4/80-positive macrophages in asbestos treated CNP mice, we asked if macrophage suppression could alone impact overall survival of this cohort. The CSF1 receptor (CSF1R) regulates multiple aspects of macrophage function as well as survival, proliferation and differentiation from monocyte precursors (Chitu et al., 2015) and CSF1R inhibition has been shown to suppress tumourigenesis and reduce resistance to chemotherapy in other cancer models (Ruffell and Coussens, 2015). Starting from day 60 post induction, mice were treated twice daily with the selective CSF1R inhibitor AZD7507 (Candido et al., 2018), or vehicle control, and maintained on drug until symptoms required euthanasia. IHC for F4/80 confirmed suppression of tumour infiltration by macrophages in all mice treated with AZD7507 (Figures 5A, B), however no survival benefit was observed (Figure 5D). RNA-SEQ analysis of tumours harvested from end-point mice treated with or without AZD7507, confirmed the on-target effects of the drug on suppressing macrophages, with bulk tumour RNA showing reduced detection of transcripts for *Csf1r* itself along with numerous other Macrophage markers including *Adgre*, which encodes the F4/80 antigen, *CD68, Mafb* and *Vsir*, encoding Vista. These data also revealed a clear reduction in multiple chemokines and chemokine receptors, suggesting broader effects on the overall tumour microenvironment (Figure 5C). We therefore asked if CSF1R inhibition could enhance the therapeutic benefit of Cisplatin/Pemetrexed doublet chemotherapy. A pilot study of chemotherapy treatment using previously published doses (Badhai et al., 2020), revealed CNP mice to be extremely sensitive to chemotherapy-induced toxicity, requiring reduction of both drugs to half the concentrations used in mesothelioma mouse model driven by triple deletion of *Bap1, Cdkn2a* and *Nf2* (Badhai et al., 2020). Starting at day 55 following induction, CNP mice induced with Lenti-Cre + asbestos were dosed with AZD7507 for 5 days, with chemotherapy also administered on the 5th day. Following a 10-day untreated recovery period, mice were again treated for 5 days with AZD7007 and a second dose of chemotherapy. Mice were then monitored until symptoms required euthanasia. As with single agent treatment with CSF1R inhibitor, no additional benefit of AZD7507 treatment was observed over chemotherapy alone. We conclude from these analyses that targeted suppression of macrophages in mice with established epithelioid neoplastic disease has no therapeutic benefit, either alone or combined with platinum doublet chemotherapy, and is therefore unlikely to show benefit in human patients with epithelioid mesothelioma.

## 4 Discussion

Chronic inflammation plays an irrefutable, albeit poorly understood, role in the development of malignant pleural mesothelioma. The persistence of durable high-aspect ratio asbestos fibres sustains the chronically inflamed state throughout the patient history, from exposure all the way through presentation, treatment, and ultimately progression to lethal disease (Donaldson et al., 2010). However, the demonstration, in complex genetically engineered mice, that targeted deletion of combinations of tumour suppressor genes commonly lost in human MPM suffices to drive mesothelioma in the absence of asbestos exposure, indicates that chronic inflammation is not required *per se* for MPM development. A plausible conjecture is thus that chronic pleural inflammation in humans creates a permissive environment for such mutations to arise but plays no further role in disease progression. Here we directly investigated this assumption using the same combination of alleles previously shown to suffice for mesothelioma development in mice (Jongsma et al., 2008). We verified that Cre-induced CNP mice are genetically proficient for development of MPM in the absence of asbestos exposure. However, we found that the additional insult of asbestos-driven chronic inflammation dramatically accelerates the onset of lethal disease. While we cannot exclude the possibility that this acceleration derives from additional asbestos-driven mutations, we note that only 4 of 15 triple heterozygous CNP mice succumbed to mesothelioma within 15 months of induction with the combination of lenti-Cre + asbestos (data not shown), which is consistent with the reported incidence for mesothelioma development in asbestos injected wild-type mice (Chernova et al., 2017): If asbestos were driving widespread genomic instability, one would have expected loss of heterozygosity at one of more of these haplo-insufficient tumour suppressor genes to manifest as increased and/or accelerated incidence of malignancy.

At the cellular level, the inclusion of asbestos did not measurably influence the tumour phenotype, although differences in the apparent growth pattern of individual lesions were noted. We detected no consistent difference in cell proliferation, expression of epithelial or fibroblast lineage markers, nor indeed in the expression of WT1, in size-matched tumours from Cre-induced CNP mice with or without exposure to asbestos. In contrast, asbestos exposure was associated with dramatically increased presence of F4/80-positive macrophage infiltration of tumour masses. Macrophages are known to be early responders to asbestos, infiltrating into the chest cavity of mice within 48hrs of asbestos inoculation (Donaldson et al., 1989). Their inability to clear high aspect-ratio fibres is thought to establish the chronically inflamed state (Murphy et al., 2012). Macrophages can have either tumour suppressive or tumour-promoting activities (Kowal et al., 2019), play major roles in recruitment or indeed suppression of other immune cell types (Biswas and Mantovani, 2010), and are associated with resistance to radio- and chemotherapy as well as to certain targeted therapies (Ruffell and Coussens, 2015). Although macrophage recruitment has been reported in GE mouse models of MPM without asbestos exposure (Badhai et al., 2020) our data suggest that tumour-infiltrating macrophages are significantly under-represented in such models. Targeted suppression of macrophages failed to improve survival in our mouse model, consistent with a similar failure as single-agent treatment in other disease models and appropriately reflecting the challenge of effective treatment of this cancer. Somewhat surprisingly, given previous reports in other cancer models (Ruffell and Coussens, 2015), macrophage suppression also failed to improve benefit of chemotherapy. It remains to be determined if macrophage suppression can be used to enhance other treatment modalities (e.g., immunotherapy) or shows efficacy against sarcomatoid disease.

The mouse model of mesothelioma presented here represents a significant refinement over previous models in 2 important ways. Firstly, our allele induction protocol did not give rise to off-target tumours, such as those reported using similar (Kukuyan et al., 2019; Badhai et al., 2020) or indeed identical (Jongsma et al., 2008) allelic mice induced with high-dose adenoviral-Cre vectors, which include sarcomas, lymphomas, and, in the case of Bap1 floxed mice, hepatocellular carcinomas. It is unclear if this difference arises from our use of a lentiviral rather than an adenoviral vector, the lower dose of virus we administered (10^7^ pfu Lenti-Cre compared with 10^9^ pfu Adeno-Cre), or a combination of both. The integration of lentiviral vectors into host cell genomes facilitates persistent expression of Cre recombinase, increasing the efficiency of floxed allele targeting and reducing the dose of virus required for effective Cre delivery. The caveat to such integration is of course that it can disrupt endogenous gene expression at the integration site. On the other hand, adenoviral vectors are inherently more stable than lentiviruses: While the tropism of lenti and adenoviral vectors would be broadly similar, the protein capsid of adenoviral vectors renders them considerably more robust than lentiviral vectors, and thus more likely to persist in circulation long enough to infect unintended cell populations (Lundstrom, 2018).

The second major distinction is the epithelioid histology of tumours arising in our mouse model, irrespective of asbestos exposure, as compared with the bi-phasic and sarcomatoid histologies of previously reported models, including those using the same allelic combination. As is the case for off-target tumour incidence, we suspect that choice and/or dose of vector may largely explain the difference in tumour histology. Specifically, adenoviral vectors are profoundly more immunogenic than lentiviral vectors and this immunogenicity may well influence the trajectory of the nascent tumour phenotype (Lundstrom, 2018). Notably, whereas loss of BAP1 is enriched in human epithelioid MPM (Hmeljak et al., 2018), the recently reported adeno-Cre induced mouse models incorporating a floxed Bap1 allele both yielded sarcomatoid phenotypes (Kukuyan et al., 2019; Badhai et al., 2020). Conversely, loss of NF2 and Tp53 are associated with the sarcomatoid subtype in human MPM (Bueno et al., 2016; Zhang et al., 2021), yet our model, which incorporates both of these tumour suppressors, yielded epithelioid tumours. These observations lead us to speculate that the microenvironment present during tumour initiation may have a stronger influence over tumour histology than the presence of any given mutation. We note that a similar phenomenon of differential tumour phenotypes arising in genetically identical models of liver cancer was recently reported: activation of the same oncogenic drivers yielded either hepatocellular carcinoma or intrahepatic cholangiocarcinoma, depending on whether cells neighbouring the tumour stem cells died via apoptosis or via necroptosis, respectively, during allele induction (Seehawer et al., 2018). It is well-established that necroptosis is a much more inflammatory form of cell death than apoptosis (Vringer and Tait, 2019)—These insights likely have direct relevance both for influencing how mesothelioma subtypes are modelled in mice and potentially for how the disease manifests in humans.

It is also clear that oncogenic mutations profoundly shape the tumour microenvironment and that different mutations will have distinct impacts on tumour-cell expression of cytokines, chemokines and other factors that regulate these effects (Hmeljak et al., 2018; Muthalagu et al., 2020; Nastase et al., 2021). In this regard it is notable that our analysis of pleural lavage from pre-symptomatic mice revealed a broad spectrum increase in leukocyte infiltration of the chest cavity as nascent tumours progressed to late stage and symptomatic disease. These data may have relevance for early detection of the transition from benign reactive neoplasia to malignant mesothelioma (Creaney et al., 2022; Crosby et al., 2022). Further work into the molecular and cellular content of pleural effusions should yield new insight into mechanisms of disease progression and potentially identify predictive biomarkers of the transition to malignancy or indeed enable accurate diagnosis of occult mesothelioma missed by histo-pathological examination of patient biopsies. Our model now provides a new platform for investigating how recurring mutations in mesothelioma influence the disease-relevant inflammatory response to asbestos and *vice versa*. The inclusion of asbestos additionally addresses a vital aspect of immediate relevance for modelling the human disease in GE mice through more accurate recapitulation of the inflammatory microenvironment that is such a crucial part of this disease. Giving particular consideration to the emerging prominence of immunotherapy in mesothelioma (Scherpereel et al., 2019; Popat et al., 2022), we believe that this model will also provide a more accurate platform for pre-clinical evaluation of new therapeutic agents and treatment strategies.

## Data Availability

The RNA-SEQ datasets presented in this study can be found at Arrayexpress: E-MTAB-12998. RNA-SEQ of epithelioid mouse mesothelioma treated with or without CSF1R inhibitor. Additional data are presented in Supplementary Material.
