# Supplementary material for: Asbestos accelerates disease onset in a genetic model of malignant pleural mesothelioma
Source: Front Toxicol. 2023 Jun 27;5:1200650. doi: 10.3389/ftox.2023.1200650 (PMC10333928; doi:10.3389/ftox.2023.1200650)
Supplement: Supplementary file 1 [file DataSheet1.pdf]

frontiers

Supplementary Material

Asbestos accelerates disease onset in a genetic model of Malignant Pleural Mesothelioma

Pooyeh Farahmand, Katarina Gyuraszova, Claire Rooney, Ximena L. Raffo-Iraolagoitia, Geeshath Jayasekera, AnnHedley, Emma Johnson, Tatyana Chernova, Gaurav Malviya, Holly Hall, Tiziana Monteverde, Kevin Blyth, Roger Duffin, Leo M. Carlin, David Lewis, John le Quesne, Marion MacFarlane & Daniel J. Murphy

*** Correspondence:**

Daniel.murphy@glasgow.ac.uk

MM2312@mrc-tox.cam.ac.uk

List of Supplemental Material

Figure S1. Examples of immunohistochemical detection of Mesothelin expression on diaphragm pleura.

Figure S2. MRI imaging of pleural effusion and mesothelioma in CNP mice induced with lenti-Cre + asbestos

Figure S3. Detection of major leukocyte populations in pleural lavage from pre-symptomatic CNP mice induced with lenti-Cre + asbestos

Figure S1

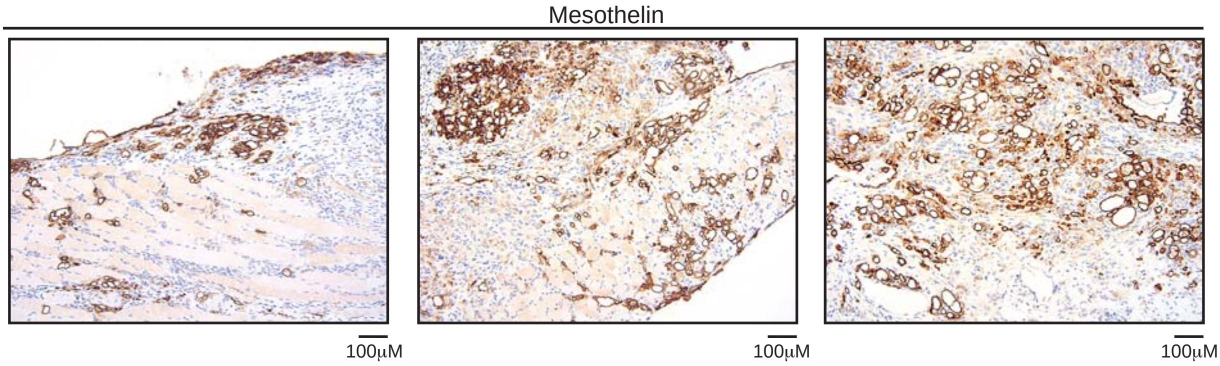

Figure S2

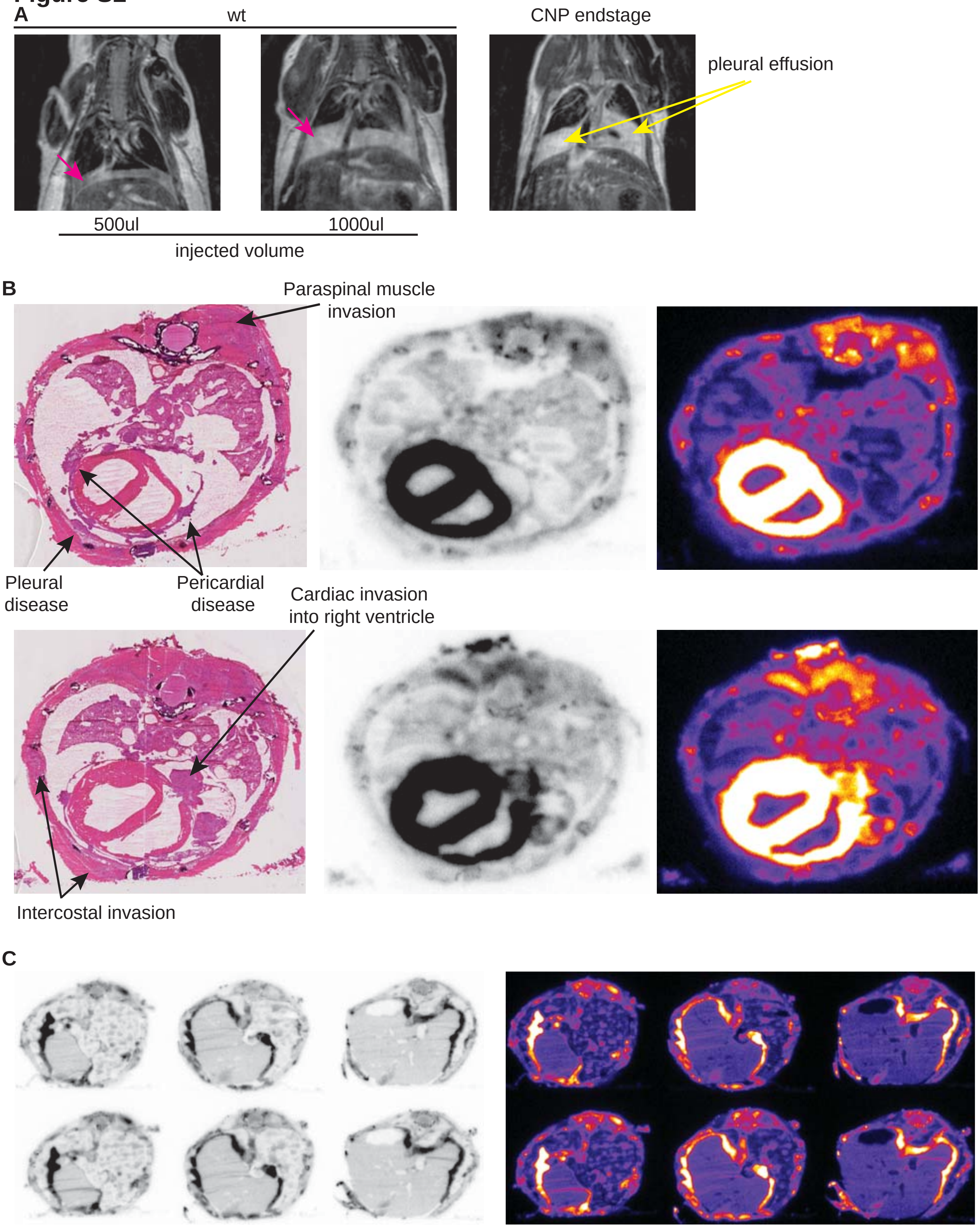
A
wt
CNP endstage
pleural effusion
500ul
1000ul
injected volume
B
Paraspinal muscle invasion
Pleural disease
Pericardial disease
Cardiac invasion into right ventricle
Intercostal invasion
C

Figure S3

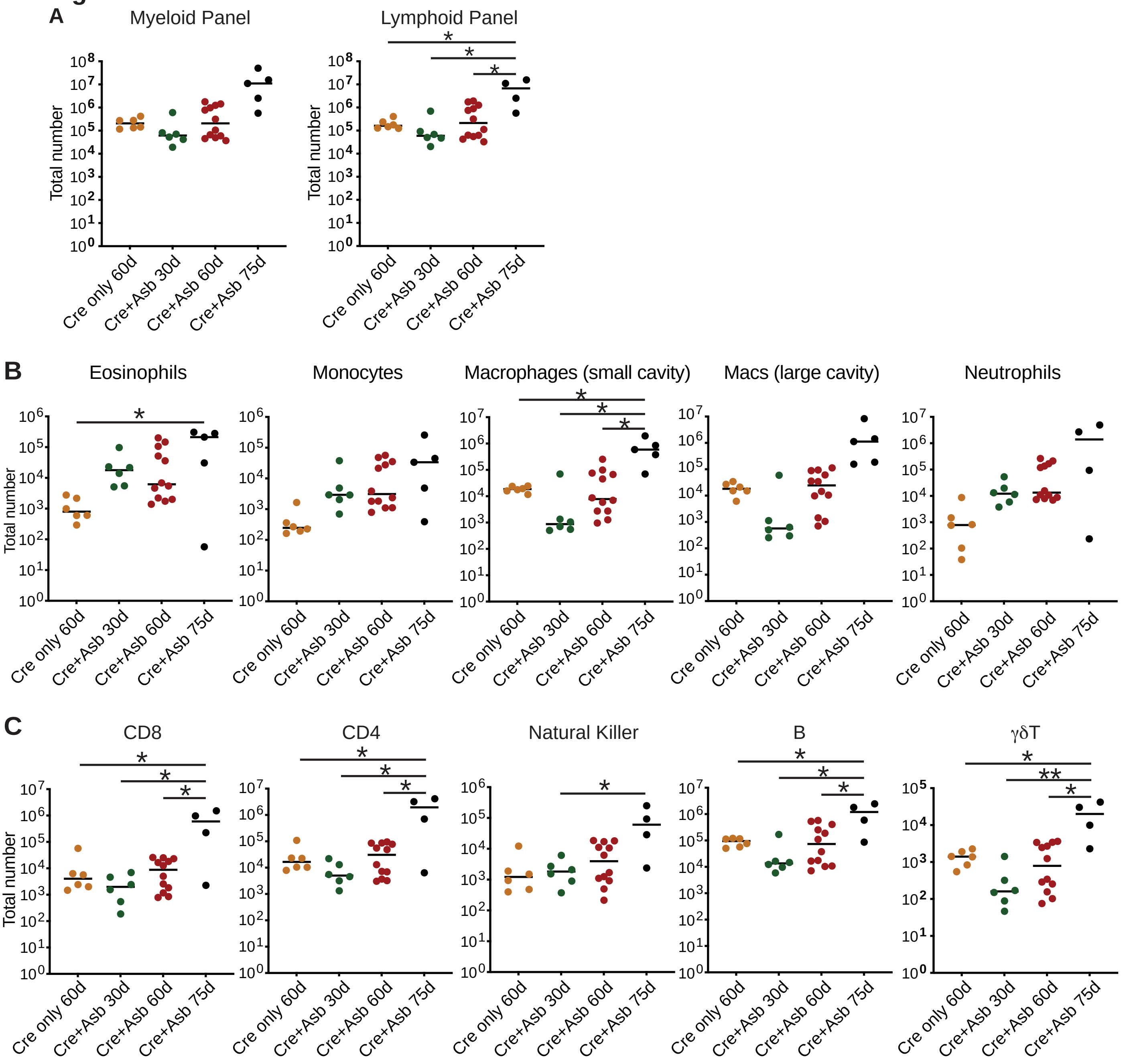
A
Myeloid Panel
Lymphoid Panel
Total number
Cre only 60d
Cre+Asb 30d
Cre+Asb 60d
Cre+Asb 75d
B
Eosinophils
Monocytes
Macrophages (small cavity)
Macs (large cavity)
Neutrophils
C
CD8
CD4
Natural Killer
B
γδT

Figure S1. Examples of immunohistochemical detection of Mesothelin expression on diaphragm pleura.

Representative images of diaphragm pleural tumours stained with anti-Mesothelin antibody (Invitrogen PA5-79698, dilution 1:500, high pH antigen retrieval). Scale bars = 100μM.

Figure S2. MR imaging of pleural effusion and mesothelioma in CNP mice induced with lenti-Cre + asbestos

A) Detection of sham (left and centre panels) or spontaneous (right panel) pleural effusion by MRI. Left to right: T2-weighted coronal/sagittal image of a wildtype mouse with a 0.5 ml intrapleural injection; T2-weighted coronal/sagittal image of a wildtype mouse with a 1 ml intrapleural injection; T2-weighted coronal/sagittal image of a terminal CNP mouse with disease associated pleural effusion. Pink arrows point to bilateral injected fluid collection; yellow arrows show pleural effusion. **B)** [^{18}F]-FDG PET/MR imaging of CNP an exemplar mouse (of 2 imaged) induced with lenti-Cre + asbestos at end-stage. Transverse sections through the chest cavity showing H&E stained tissue (left panels) and [^{18}F]-FDG detection by autoradiography in brightfield (centre panels) and false-colour (right panels) of corresponding whole mount sections. Invasive, pericardial and pleural disease indicated by arrows. **C)** Autoradiography of [^{18}F]-FDG detection showing extent of pleural disease in an exemplar mouse in brightfield (left) and false colour (right). Note detection of pleural effusion in the 3rd section from left (white area in brightfield, black area in false colour).

Figure S3. Detection of major leukocyte populations in pleural lavage from pre-symptomatic CNP mice induced with lenti-Cre + asbestos

A) FACS quantification of total WBC count/ml of pleural lavage from CNP mice induced with lenti-Cre alone (N=6; day 60) or lenti-Cre + Asbestos, harvested at 30 (N=6), 60 (N=12), or 75 (N=5) days post induction. Left panel shows total CD45+ cell counts per mouse, stained with the Myeloid marker panel; right panel shows total CD45+ cell counts per mouse, stained with the Lymphoid marker panel. B) Major Myeloid cell populations quantified from (A). C) Major Lymphoid populations quantified from (A). ** denotes $P<0.01$; * denotes $P<0.05$, 2-way ANOVA with post-hoc Tukey test.
